# Role of hydrostatic pressure and wall effect in solidification of TC8 alloy

**DOI:** 10.1038/s41526-019-0083-2

**Published:** 2019-10-11

**Authors:** Xinghong Luo, Yaya Wang, Yang Li

**Affiliations:** CAS Key Laboratory of Nuclear Materials and Safety Assessment, Institute of Metal Research, Chinese Academy of Sciences, 72 Wenhua Road, Shenyang, 110016 China

**Keywords:** Materials science, Condensed-matter physics

## Abstract

The solidification experiments of TC8 alloy under both microgravity and normal gravity were conducted using a drop tube. The solidification microstructure were found composed of fine equiaxed grains formed at early stage and bigger elongated grains formed at later stage. Between the two kinds of grains a curved transition interface was observed in 1g sample, while that in μg sample was almost flat. Generally, the amounts and aspect ratios of the grains are larger, and the grain sizes are smaller in 1g sample. Besides, no visible element macrosegregation occurred in both samples. The results suggest that the solidification velocities of the samples were rapid, and consequently the convection effect and solute transport effect caused by gravity had little influence on the solidification microstructure. Therefore, the solidification process was mainly controlled by thermal diffusion, and hydrostatic pressure and wall effect played a great role in it.

## Introduction

With the continuous development of aerospace technology and advanced material preparation technology, people are looking for the perfect combination of the two technologies to serve the future deep space exploration and interstellar navigation. Space additive manufacturing technology is expected to be one of them.^1–3^ With this technology, astronauts can make whatever parts they need in situ, without having to spend precious payload resources to carry them directly from the ground. Currently, NASA, ESA, and other space agencies are working on developing related technologies. As a kind of typical light metal, titanium alloy has a series of excellent properties, such as high specific strength, good machining performance, strong corrosion resistance, etc., and is widely used in the aerospace field,^4–6^ so it is expected to be a candidate material for additive manufacturing in space. Microgravity effect exists in space environment, where buoyancy convection, hydrostatic pressure, sedimentation phenomena disappear basically, which will have important impacts on the solidification of alloys,^7,8^ resulting in obvious changes in dendritic, eutectic, monotectic, and other microstructure.^9–11^ Different from usual columnar solidification structure, the solidification structure of duplex titanium alloy is usually equiaxed polycrystalline structure.^12^ However, few works were reported on the solidification behavior of titanium alloy and polycrystalline structure in microgravity environment. In order to better understand the effect of space microgravity environment on the solidification behavior of titanium alloy, so as to provide necessary technical support for its space additive manufacturing, it is necessary to conduct an experimental study on it in advance. In view of this, one of commonly used titanium alloys, TC8, was selected in this paper to conduct a comparative study on its solidification behavior in microgravity and gravity environments with a 50-m-high drop tube,^11,13–15^ so as to obtain the specific influence of microgravity effect on the solidification of titanium alloys.

## Results

**Fig. 1:**
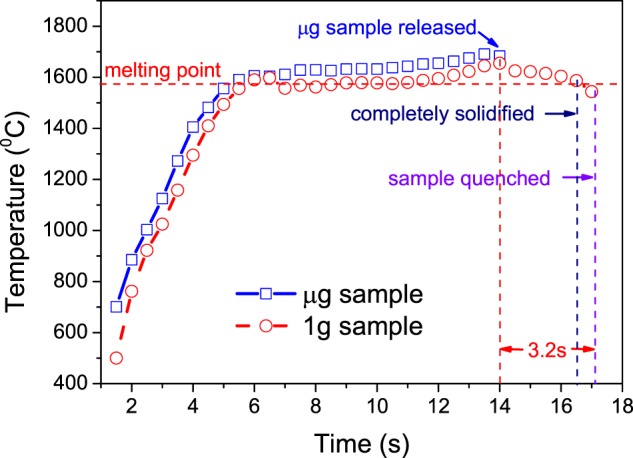
Temperature–time profiles of μg sample and 1g sample. The solid blue curve with square symbol shows the temperature–time profile of μg sample. The dashed red curve with circle symbol shows the temperature–time profile of 1g sample

Figure 1 shows the temperature vs. time of the sample tops during the experiments. Because the temperature during the falling process could not be monitored, only the temperature before release of the μg sample was recorded. It can be seen that the curves of heating and melting phases of both samples are highly coincident, indicating that μg sample and 1g sample have same heating history. Given that the external cooling environments of the both were the same room temperature and vacuum environment, it can be reasonably speculated that the cooling environment of the both were basically the same, which ensured that the gravity level was the only variable during the experiments. According to the complete temperature–time curve of 1g sample, it can be seen that the top temperature of the sample dropped below the melting points after the power was turned off for about 2.5 s (within 3.2 s), that is, the solidification of the samples had finished before quenching in the silicon oil.

**Fig. 2:**
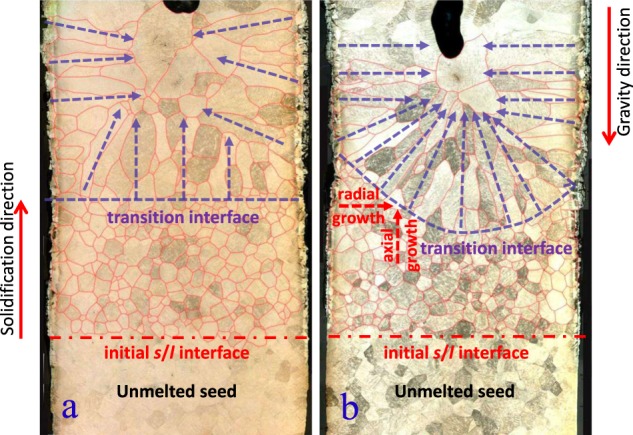
Longitudinal section microstructure of the samples. **a** μg sample and **b** 1g sample. From the bottom up, the red dash dot lines indicate the positions of the initial solid–liquid interfaces, the blue dashed lines indicate the positions and shapes of the transition interfaces, and the blue dashed lines with arrows indicate the growth directions of the grains. The diameter of the samples is about 6 mm

Figure 2 shows the solidification microstructure in longitudinal sections of the samples. It can be seen that the solidification structures of both 1g sample and μg sample are polycrystalline structures, and the grain size increases with the increase of the distance from the initial solidification interface. Morphologically, there are two kinds of grains, one is the equiaxed grain growing upwards from bottom at the early stage of solidification, and the other is the elongated grain growing with obvious orientation at the late stage of solidification. Between the two kinds of grains, a transition interface could be observed in each sample. In μg sample, the interface is almost flat; while in 1g sample, the interface is curved, like a basin. Below the interface, the solidification structure of both 1g sample and μg sample are fine equiaxed grains, no evident difference between them could be seen, except that the area below the transition interface is larger in μg sample than in 1g sample. Above the interface however, the grain size, morphology, and orientation are significantly different between 1g sample and μg sample. In μg sample, the elongated grains first grew upward along the direction perpendicular to the flat transition interface, especially in the center of the sample. Then, with the solidification, the direction of grain growth gradually shifted from vertical upward to tilt toward the center. Finally, near the top of the sample, the grain growth direction became radial or even slightly inclined downward. Nevertheless, in 1g sample, the elongated grains basically grew towards the normal direction of the basin-like transition interface, and then gradually shifted to radial direction, after that, this pattern kept on even in the vicinity of the top of the sample.

**Fig. 3:**
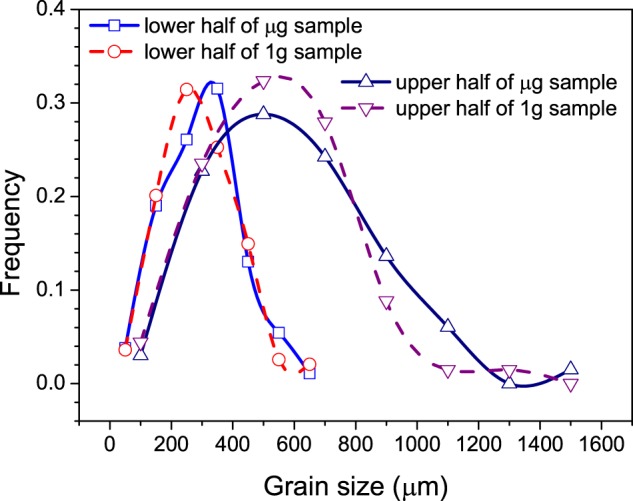
Grain size distribution frequencies of μg sample and 1g sample. The blue curve with square symbol shows the size distribution frequency of the grains below the transition interface in μg sample, the red curve with circle symbol shows the size distribution frequency of the grains below the transition interface in 1g sample; The navy curve with up triangle symbol shows the size distribution frequency of the grains above the transition interface in μg sample, the purple curve with down triangle symbol shows the size distribution frequency of the grains above the transition interface in 1g sample

Considering that the morphology and the size of the grains above and below the transition interface are quite different, the distributions of their diameters were statistically analyzed separately by equal area circle method, and the results are shown in Fig. 3. The statistical data on number, size and morphology of the grains above the transition interface in both samples are also listed in Table 1.

**Table 1:** Statistical data on grains in upper half part of μg and 1g samples

Sample	Grain number per unit area (mm^−2^)	Grain size (μm)			Aspect ratio		
		Ave.	Max.	Min.	Ave.	Max.	Min.
μg	3.126	595	1512	187	2.385	6.177	1.054
1g	3.807	543	1202	167	2.966	7.345	1.058

Obviously, the average sizes of the grains in the equiaxed grain zones are much smaller than those in the elongated grain zones in both μg and 1g samples, and the average grain sizes in both the equiaxed grain zone and the elongated grain zone in μg sample are larger than those in 1g sample. In detail, the distribution peak of grain size in the equiaxed grain zone in μg sample locates on the right of that in 1g sample, indicating that most of the grains in that zone in μg sample are larger than those in 1g sample, but the difference is not big. In the elongated grain zone, there are fewer small grains (<800 μm) and more big grains (>800 μm) in μg sample than in 1g sample, making the average grain size larger than that of 1g sample. On the other hand, the elongation of grains in 1g sample is greater than that in μg sample, according to the data shown in Table 1, implying a sign of stronger oriented growth.

**Fig. 4:**
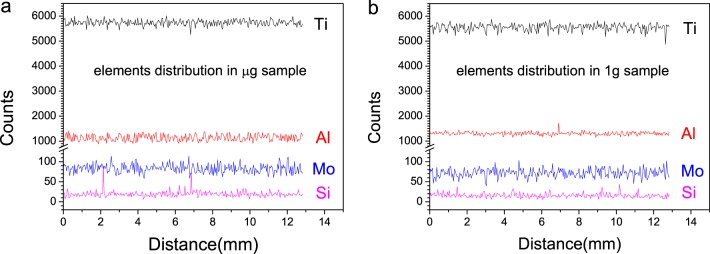
Line scanning analysis results of the elements distribution along solidification direction. **a** μg sample and **b** 1g sample. From the top down, the black spectral lines represent the distributions of Ti element, the red spectral lines represent the distributions of Al element, the blue spectral lines represent the distributions of Mo element, and the pink spectral lines represent the distributions of Si element

The axial element content distribution from the remelting interface upward to the vicinity of the shrinkage cavity in μg sample and 1g sample are shown in Fig. 4. No obvious change of the element content with the distance can be seen in both samples, that is, no macroscopic segregation occurred. Besides, there is no significant difference between the composition distribution in μg sample and that in 1g sample.

## Discussion

Generally, one of the most important differences of normal gravity from microgravity environment is the existence of buoyancy convection, which is evoked by the uneven density inside melt due to temperature gradient or concentration gradient. It usually has a significant impact on solidification structure and solute distribution.^11,13,15–19^ In this work, however, things were different. TC8 alloy is an alloy with high melting point. Due to the huge difference between the high heating temperature (around 1650 °C) and low ambient temperature, as well as the existence of the unmelted cold end, the average solidification rate could be fast. It was roughly estimated to be about 2.6 mm/s for the both samples, by measuring the distance from the initial solidification interface to the top shrinkage cavity in combination with the solidification time shown on the temperature–time curve. This velocity was beyond the average convection velocity and solute diffusion rate in melt, which was reported in the order of 1 mm/s and 10^−2^–10^−1^ mm/s, respectively.^20,21^ As a result, the impact of buoyancy convection on the solidification microstructure and solute distribution could be neglected,^11^ which was partially testified from one side by the elements distribution analysis results. Another result led by the high solidification velocity was the formation of equiaxial polycrystalline structures instead of common columnar structures.^22^

Actually, besides buoyancy convection, the difference between microgravity environment and normal gravity environment includes hydrostatic pressure and wall effect as well. They both have influence on the heat dissipation, especially through crucible wall, during solidification. In addition, the wall effect may also induce heterogeneous nucleation.^23^ Therefore, under the solidification condition in this paper, the heat in the melt had two dissipation channels, one was the axial dissipation through the solid, and the other was the radial dissipation through the crucible wall. Concretely, according to the results, in initial solidification phase of the samples, because of the strong axial heat dissipation effect of the unmelted cold end, the direction of heat flow in the melt near the solid–liquid interface was downward perpendicular to the solid–liquid interface; at the same time, large temperature difference also resulted in large temperature gradients near the solid–liquid interface, the combination of the both caused the melt to solidify upward at a very fast speed and form fine equiaxial polycrystalline structures. In this phase, hydrostatic pressure was not inactive, although its effect was relatively weak. Under hydrostatic pressure, the melt in 1g sample contacted with the crucible wall more tightly, making it easier to dissipate heat via the crucible wall and to nucleate heterogeneously on the crucible wall, and consequently producing a higher nucleation rate and a higher growth rate. In μg sample however, without hydrostatic pressure, the contact between the melt and the wall was weak or even, to some extent, detached if the wettability between the melt and the wall was not good enough. That may explain why the grain number density was higher and grain size was smaller in 1g sample than in μg sample. After the rapid solidification in the initial phase, the axial temperature gradient in front of the solid–liquid interface decreased gradually as the temperature of the solid end increased, and the nucleation and growth rate slowed down gradually. In this case, the effect of hydrostatic pressure and wall was gradually highlighted. In another words, the radial temperature gradient and wall nucleation came into play. They let the nucleation rate and growth rate near the surface of 1g sample to be competitive with those at the center of the sample, and the result of the competition between the radial inward growth from the wall and the upward growth along the axial was to bend the macroscopic solid–liquid interface to form a basin-like structure shown in Fig. 2b. Different from this, such effect was minimal in μg sample, and the macroscopic solid–liquid interface remained flat.

When the solidification process was half over and the solid–liquid interface was getting closer and closer to the upper surface of the melt, the temperature of the solidified solid was getting higher and higher due to absorption of heat dissipation from melt and latent heat of solidification, and the axial temperature gradient in the melt became smaller and smaller. In this situation, axial heat dissipation through the solidified solid was becoming difficult, and that through the outer surface of the melt gradually occupied a dominant position, which made the heat dissipation direction in the melt deflect from vertical downward to radial outward gradually. This was especially true in 1g sample, the occurrence of deflection was obviously earlier and the degree was higher than those in μg sample, respectively. As the nucleation rate and growth rate of the melt decreased further with temperature gradient, the solidified structure gradually changed from the fine equiaxial grains in the initial phase to the elongated grains growing in the opposite direction of heat dissipation. Comparatively, the grain size in μg sample was bigger and the aspect ratio of grain in 1g sample was larger, suggesting that the nucleation and growth rates were slower in μg sample and the heat flow in 1g sample was stronger. The reason for these differences was closely related to hydrostatic pressure. In μg sample, due to lack of hydrostatic pressure, the melt surface tension played a prominent role. The melt had a poor wetting on the crucible and the heat exchange with the surrounding area was weak. Therefore, the temperature gradient in the melt was lower, leading to a lower nucleation rate and growth rate.^24^ In 1g sample, on the other hand, with the assistance of hydrostatic pressure, the melt and the crucible wall were wetting better, the heat in melt diffused to the surrounding environment more easily, which produced higher temperature gradient and promoted heterogeneous nucleation,^23^ leading to higher nucleation rate,^25,26^ growth rate and more significantly oriented growth. This indicates that, with the solidification rate slowing down, the change of solidification process caused by the difference of hydrostatic pressure was further highlighted.

Until close to the end of solidification, under hydrostatic pressure, the elongated grains in 1g sample basically maintained the growth trend along the radial directions towards the sample center. However, the grain growth direction in μg sample showed a tendency of continuous upward deflection. At this time, without hydrostatic pressure, the surface of μg sample had most likely detached from the crucible wall completely, due to volume shrinkage during solidification and surface tension effect, and became free surface. Then the grains grew in a direction perpendicular to the sample surface towards the sample center.

To sum up, in addition to the influence of the buoyancy convection and sedimentation on the concentration field, temperature field and flow field in melt during solidification process, and thus on the solidification structure of alloys, the hydrostatic pressure caused by gravity will also change the heat diffusion in melt due to the influence on interface effect, so as to have a significant impact on solidification structure. Furthermore, even when the solidification velocity exceeds the convective velocity, rendering it ineffective, the hydrostatic pressure can still affect the solidification structure. Therefore, the effect of hydrostatic pressure should not be ignored when analyzing the influence of gravity on the solidification structure of materials.

## Methods

**Fig. 5:**
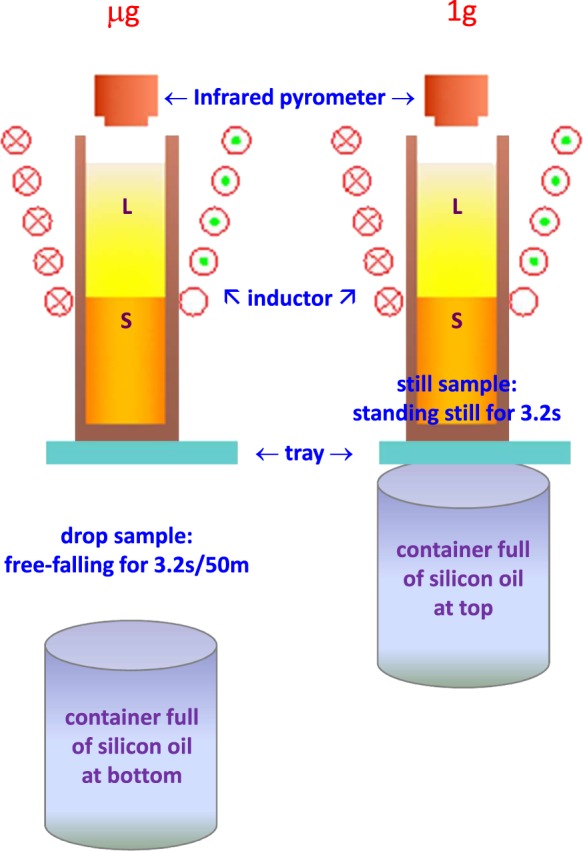
Schematic diagram of the experimental setup

Rod samples with diameter of 6 mm and length of 28 mm were machined from extruded rod of TC8 alloy with chemical composition of Ti–6.33%Al–3.46%Mo–0.27Si(wt%). The experimental facility is a 50-m-high drop tube with a diameter of 150 mm, which can be evacuated down to 1 × 10^−4^ Pa, and therefore, supply a microgravity environment at an acceleration level down to 10^−6^*g*_0_ for about 3.2 s. A schematic diagram of the experimental setup is shown in Fig. 5. At the top of the drop tube, an induction coil and a 10 kW semiconductor high-frequency induction power supply are equipped. Besides, a monochrome infrared pyrometer is installed at the top of the drop tube to monitor the temperature of samples from their top ends. The experimental procedures are as follows: The sample is loaded into a corundum crucible with inner diameter of 6 mm, height of 30 mm, and wall thickness of 1 mm, and placed in the center of the induction coil in the vacuum chamber at the top of the drop tube. A removable tray is used to support the crucible from its bottom. Adjust the height of the sample in the coil so as to let only the upper end of the sample be heated to melt and keep the lower end of the sample as solid. Switch on the power supply to heat the sample for some seconds until its upper end is melted, then switch off the power supply and remove the tray simultaneously to release the sample. During its free fall in the tube, solidification under microgravity from the unmelted solid end of the sample takes place, until it falls down to the bottom of the tube, where a container full of silicon oil is placed to quench and collect the sample. For comparison purpose, same experiment on same sample is conducted without free fall. Specifically, the sample is not released immediately after melting but kept still on the tray for 3.2 s, then the tray is removed and the sample was allowed to quench in silicon oil just below it. In this case, solidification under normal gravity from the unmelted solid end of the sample takes place. For the convenience of description, in this paper the drop sample is referred to as μg sample, and the still sample is referred to as 1g sample.

The experimental samples were cut longitudinally along their central axes. The microstructure on the longitudinal sections of the samples were observed and photographed using a MEF4A metallographic microscope after sample grinding, polishing, and etching with 2%HF + 5%HNO_3_ + 93%H_2_O (vol%) solvent. Since the grain structure in the solidified microstructure was not clearly displayed, the grain boundaries in microstructure were highlighted by image editing software. Then the Image pro plus 6.0 analysis software was used for statistical analysis of the processed images, and the size distributions, numbers, and aspect ratios of the grains were obtained. In addition, the composition distributions along the central axes on the longitudinal sections of the samples were analyzed by line scanning with electron probe X-ray microanalysis (EPMA).

### Reporting summary

Further information on research design is available in the Nature Research Reporting Summary linked to this article.

## Supplementary information

Reporting Summary Checklist

## Data Availability

The datasets generated and analyzed during the current study are available from the corresponding author on reasonable request.
